# Effects of Strength Training Program and Infrared Thermography in Soccer Athletes Injuries

**DOI:** 10.3390/sports6040148

**Published:** 2018-11-19

**Authors:** Pedro Menezes, Matthew R. Rhea, Carlos Herdy, Roberto Simão

**Affiliations:** 1 Escola de Educação Física e Desportes (Departamento de Ginástica), Universidade Federal do Rio de Janeiro, Av. Carlos Chagas Filho, Cidade Universitária, Ilha do Fundão, Rio de Janeiro 21.941-590, Brazil; carlosherdy@gmail.com; 2 Department of Human Movement, A.T. Still University, 5850 E. Still Circle, Mesa, AZ 85206, USA; matt.racerx@gmail.com

**Keywords:** periodization, treatment outcome, performance, contusions

## Abstract

The purpose of this investigation was to examine the effects of a periodized strength training program and the use of infrared thermography (IRT) in injuries mapping in under 20-year-old (U-20) soccer players. In this study, 26 professional soccer players participated in strength training (ST) twice a week and were tested with IRT consistently across the 1-year. Strength, vertical jump, heat differences and injuries were tracked and analyzed. Results: 69 injuries occurred during 12 months of tracking; most identified injuries were: contusions, sprains, strains to the thigh (n = 16), ankle (n = 15) and knee (n = 12). Differences (>7 °C) in IRT patterns were noted among injured and non-injured athletes. Significant improvements in strength (*p* < 0.005) were found for vertical jump, bench press, front lat pull down, shoulder press, leg press, leg curl and squat. Number of injuries decreased from 23 (33.3%) to 14 (20.3%) when early year rates were compared to late year (*p* < 0.005). Combined ST and IRT represent useful strategies for reducing injuries among U-20 soccer players.

## 1. Introduction

Injuries are a major adverse event during a soccer player’s career, as they require medical treatment and rehabilitation, thus interrupting a player’s activity for a period spanning from few weeks to several months, often with severe physical and psychological sequels [1,2,3,4,5,6]. The most common of soccer injuries are sprains, strains and contusions occurring most frequently at the ankle, knee joints, and in the muscles of the thigh and calf [4,5,6,7,8]. Between 8% and 33% of all injuries during the competitive phase are classified as overuse injuries [6,7,8].

Further study of soccer injuries demonstrates that a common cause is insufficient strength and collision with another player [1,4,5,6]. Between 13% and 27% of all injuries are related to foul play [1]. The percentage of non-contact injuries varies from 25% to 57%, mainly occurring during running and turning. Approximately, 20–25% of all injuries are re-injuries of the same type and body location [8]. Preceding injury was noted as a major risk factor for future injury [7,8,9]. Most researchers have reported risk factors for soccer injuries and discussed options for prevention [1,4,5,6]. It is mandatory for experts to determine the major risk factors for injuries, thus enabling targeted intervention efforts to prevent injuries from occurring [1,4,7]. More research in this area is needed [4,8].

Some plausible etiological factors correlated to injury in soccer include: (1) unbalanced flexibility of the muscles, (2) muscle weakness and/or insufficient strength, (3) inefficient muscle coordination, (4) deficient warm-up and stretching before activity, and (5) premature return to activity (before complete rehabilitation from a previous injury) [7,8,10]. Under a theoretical model, it was suggested that a combination of abnormalities related to strength, flexibility, warm-up method, and fatigue play important roles in increasing the risk of injury [1,7,8].

Strength training among soccer players can improve players’ specific and relevant athletic abilities inherent in their sport. Many soccer training programs merge different exercise methodologies (e.g., strength training, plyometric training, and sport-specific agility), enabling strength and power development which transfers to athletic activities due to both the neural and morphological adaptations [10,11,12,13].

Soccer consists of a varied range of motor skills that embraces not only both breaking and quick forces but also distinct strength modes and velocities that require the all force-velocity potential of the neuromuscular system [10,12,13]. Several studies have demonstrated that just a few weeks of resistance training (ST) may provide the increase of power, strength and functional performance, due to both neural and muscular adaptations, and resulting in the reduction in sport injuries [11,12]. However, to the best of our knowledge, there are no research that have investigated the impact of 1-year periodized strength training program on the occurrence and re-occurrence of injury in (U-20) professional soccer players.

Another method that has begun to receive attention regarding injury prevention is infrared thermography (IRT) [13,14]. One of the most beneficial contributions of IRT to sports may be within the ambit of preventive medicine. A recent study examined tendonitis in race horses and IRT was able to detect altered or imbalanced heat patterns two weeks before clinical evidence of swelling, pain and lameness [8]. Other research has demonstrated that thermal images from the two sides of the body are usually symmetrical and an asymmetry of more than 0.7 °C can be defined as abnormal [4,8]. These abnormalities may indicate a problem and foster early detection of abnormal changes in the tissues that may precede an injury [13,14,15].

IRT is a tool of major importance as it allows a non-invasive and non-radiating analysis of physiological functions related to the control of skin-temperature. This technique detects infrared radiation that can be directly associated with the temperature distribution of a determined body region [14,15] Commonly, an injury causes modifications in blood flow which, in turn, can influence skin temperature. Inflammation results in hyperthermia, whereas degeneration, decreased muscular activity and poor perfusion may lead to a hypothermic pattern [14].

The hypothesis of the present study was that the enhancement of muscle strength prevents the occurrence of injure/re-injury and IRT represents useful strategies for mapping injuries.

To sum up, the present prospective cohort study was designed to examine the incidence and the occurrence of injuries in (U-20) soccer players performing regular ST following by IRT mapping throughout a 1-year season.

## 2. Materials and Methods

### 2.1. Experimental Approach to the Problem

U-20 players trained two times per week and each session consisted in both technical training and physical conditioning. Those training sessions took from 60 to 120 min. Typically, the team schedule embraced playing their competition on a Saturday and training on Monday, Tuesday or Thursday. Sessions involved sprint training, speed endurance training, high intensity aerobic training and strength training. Usually, players would rest on Sundays and, before games on Fridays, they were submitted to a light technical session. The in-season phase consisted of eight league matches in a 12-week period. Players participated in a range of 4–11 matches per player due to squad rotation and player availability.

All injuries resulting in a player being unable to fully participate in training or match play (i.e., time-loss injuries) were recorded. One player remained classified as “injured” until the team medical staff authorized entire participation in training and availability for match selection. The team medical staff recorded injuries by using a standard form in which information about diagnosis, nature and circumstances of injury were reported.

Preceding the beginning of training period, and every 3 months thereafter, the players performed the following tests:

(1) Vertical jump performance test (VJ), with hands on the hips, starting from a static standing position and with their legs straight during the flight phase of the jump [16]. Participants were instructed to jump as high as possible. The calculation of VJ height was developed using the iPhone App, named “My Jump”, which includes a 120 Hz high-speed camera, at 720 pixels; [16].

(2) One repetition maximum (1 RM) testing. The 1 RM testing began with a warm-up at 50% of the predicted 1 RM. After five minutes of rest, each subject was encouraged to perform one repetition with a heavier load. If the attempt was successful, the load was increased, and the attempt repeated [17]. After 72 h a second visit occurred and the 1 RM test was repeated, with the highest successful lift being recorded as the 1 RM. The exercises performed were the bench press (BP), front lat pull down (LPD), shoulder press (SP), leg press (LP), leg curl (LC) and squat (SQT). The 1RM assessments were divided over a four-day period. On the first- and third-day LP, LC and SQT were tested and retested, on second- and fourth-day BP, LPD and SP were performed [17].

### 2.2. Subjects

The entire soccer team, twenty-six male professionals (U-20) age soccer players, agreed to participate and represented the sample of this study. Subject characteristics included: 18 ± 1 years; 1.81 ± 0.08 cm; 76.3 ± 9.8 kg; body fat (%) 12 ± 1.9; Power Member Inferiors (PMI) 4300 ± 381 Watts. The study was approved by an Institutional Review Board and Ethics Committee for research with human subjects, protocol CAAE 09957813.4.00005108. We provided the research details to the team and their informed consent was secured prior to the commencement of the study. All participants played for the same team in the Brazilian Football League. Exclusion criteria included: (1) smoking history during the previous three months; (2) presence of any cardiovascular or metabolic disease; (3) systemic hypertension (≥140/90 mmHg or use of antihypertensive medication); (4) use of anabolic steroids, drugs or medication with potential impact on physical performance (self-reported).

### 2.3. Procedures

#### 2.3.1. Infrared Thermography (IRT)

An IRT image of the leg, femoral quadriceps and biceps of each athlete was obtained at the beginning of the study and the start of the match season. Images were collected throughout the season, 24–48 h after each official match [18]. Additionally, if pain was reported or thermography temperature was asymmetrical (>0.7 °C) images were taken every day until symptoms disappeared. The IRT was taken in an acclimatized room with temperature of 23 °C. The athlete remained for 15 min in the room for thermal balance before the image acquisition process began. The following material was used: a thermography camera (Flir Systems Inc., Wilsonville, OR, USA; model FLIR ONE PRO), a computer (with specific thermography images acquisition and processing—Flir tools) and a digital thermo-hygrometer (Minipa^®^ model MT241) for room temperature and humidity monitoring.

The distance between the subject and the camera was standardized at two meters and the index of human skin emissivity was set to 0.98. Analyze of the body regions of interest (ROI) were selected by a drawing rectangular area by the software—Flir tools which provided us with the average and maximum temperatures from each analyzed ROI [13,14,15]. Selection of the ROI utilized 5 cm above the upper border of the patella and groin line for the thigh, and for the leg, 5 cm below the lower border of the patella and 10 cm above the malleolus.

The highest and lowest temperatures mean temperatures and standard deviation of the selected region of the muscle were collected. Significant asymmetry of more than 0.7 °C from two sides of the body was defined as abnormal.

The IRT camera used a thermal resolution of 160 × 120 pixels, with sensors that allow measurement of temperatures ranging from −20 °C to 120 °C. This camera has the sensitivity to detect differences in temperature lower than 0.03 °C and accuracy of ±1 °C of absolute temperature, according to the manufacturer’s guidelines.

#### 2.3.2. Training Protocol

Periodized ST in four mesocycles was used in this study with an emphasis on upper-body, lower body, and total body muscular power enhancement. Five exercises were performed in each session. Supervised ST was conducted 2 times per week during each mesocycle, and the exercises performed, volume, and intensity were dictated across the mesocycles for all athletes.

The ST consisted of 2–4 sets of 6–20 RM, witch auto suggest recovery between the training sets and exercises [19], performed twice a week, as described below.

1st Phase—12 Weeks

▪ 2 weeks/3 sets × 12–15 RM.
▪ 2 weeks/3 sets × 8–10 RM.
▪ 2 weeks/3 sets × 10–12 RM.
▪ 2 weeks/4 sets × 8–10 RM.
▪ 3 weeks/4 sets × 15–18 RM.
▪ 1 week/4 sets × 4–6 RM.

2nd Phase—12 Weeks

▪ 3 weeks/3 sets × 8–10 RM.
▪ 2 weeks/3 sets × 10–12 RM.
▪ 2 weeks/3 sets × 8–12 RM.
▪ 2 weeks/4 sets × 8–10 RM.
▪ 2 weeks/4 sets × 15–20 RM.
▪ 1 week/4 sets × 6–8 RM.

3rd Phase—12 Weeks

▪ 2 weeks/4 sets × 12–15 RM.
▪ 2 weeks/4 sets × 8–10 RM.
▪ 2 weeks/4 sets × 10–12 RM.
▪ 2 weeks/4 sets × 8–10 RM.
▪ 3 weeks/4 sets × 15–20 RM.
▪ 1 week/2 sets × 6–8 RM.

4th Phase—12 Weeks

▪ 2 weeks/3 sets × 12–15 RM.
▪ 2 weeks/4 sets × 8–10 RM.
▪ 2 weeks/3 sets × 10–12 RM.
▪ 2 weeks/4 sets × 6–8 RM.
▪ 3 weeks/4 sets × 15–20 RM.
▪ 1 week/2 sets × 6–8 RM.

#### 2.3.3. Statistical Analysis

Repeated Measures Analysis of Variance test (Two-way ANOVA) was applied to compare the strength gain over the following parameters: 1 RM test, vertical jump, body free mass, body fat and outcome days performed on each three-month cycle for one year. In all cases, the post-hoc Bonferroni was used. Alpha was set at *p* < 0.05 and all analyses were performed with the SPSS (Version 16.0 for Windows; SPSS Inc., Chicago, IL, USA). Injury incidence was calculated as the number of injuries per 100 played hours and evaluated across the same phases of training. The significance level was set at *p* < 0.05. Cox regression analysis was applied on IRT for injuries control and prevention.

## 3. Results

Table 1 shows operational definitions used in this study for clarification of injury depiction. All injuries resulting in a player being unable to participate fully in training or match play (i.e., time-loss injuries) were recorded. The player was classified as injured until the team medical staff allowed full participation in training and availability for match selection.

A total of 69 injuries were recorded in the 12-month period. Of those, 16 occurred in the thigh, 15 in the ankle, 12 in the knee, representing the most common injuries. Table 2 shows the number and location of injuries. Re-injuries were accountable for 15% of all the injuries sustained during the study period. Of the 11 re-injuries that were documented, 8 were either strains or sprains. There were many instances where a player had already sustained one injury during the season, which was subsequently followed by an injury to the same anatomical location in the next injury episode. Re-injuries were found to be more severe than the previous injury with the number of missed training days averaging 13.1 compared with 8.2 days for the initial injury (*p* < 0.005).

Injuries were defined in 41 cases as grade 1; in 24 events (35%) as grade 2, and in four events (5%), as grade 3. Table 3 showed the type of injuries. In addition, 4 types of injuries were identified: 23 muscular (33.33%), 23 articular (33.33%), 15 trauma (21.74%), and 8 tendinous (11.60%). Table 4 and Table 5 show the classification and the phases of injuries, respectively.

**Figure 1:**
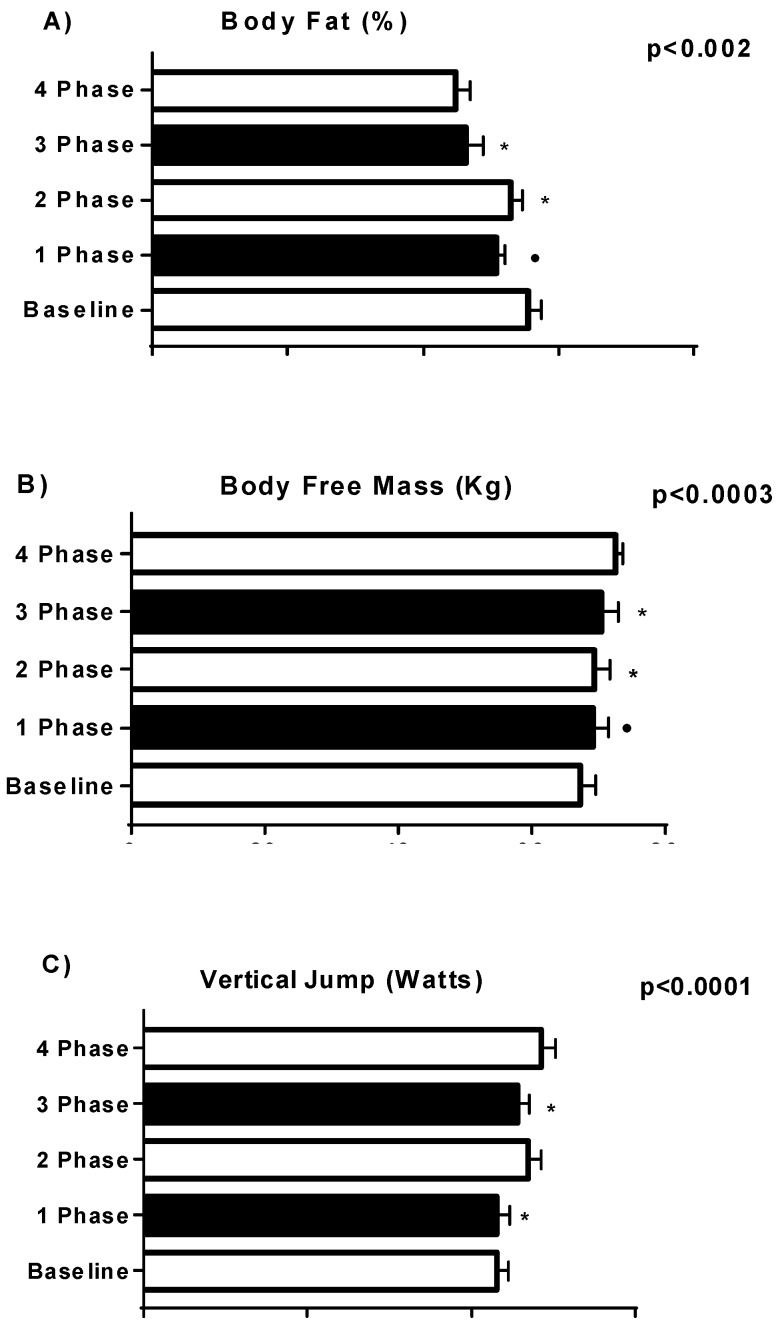
Changes in physiological and functional parameters resulting from strength training. * difference between phases. • difference between phase and the baseline.

**Figure 2:**
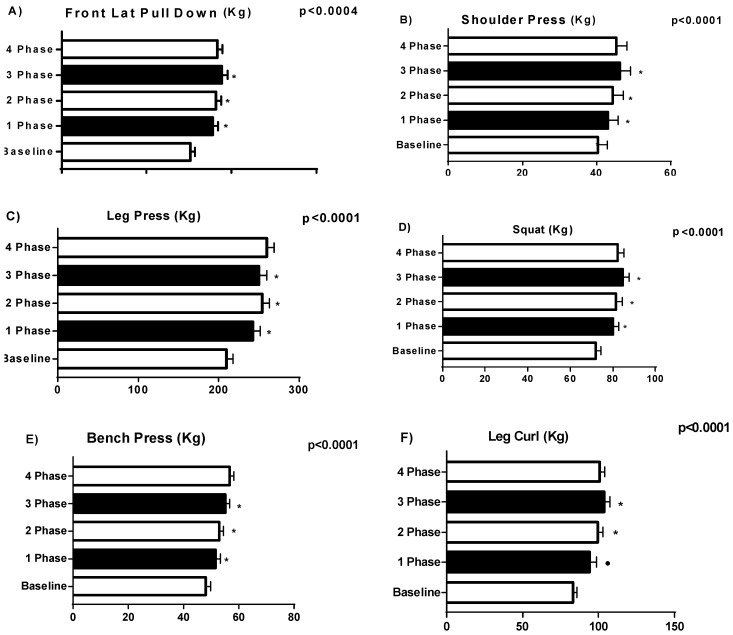
Changes in physiological and functional parameters resulting from concurrent strength and endurance training. * difference between phases. • difference between phase and the baseline.

**Figure 3:**
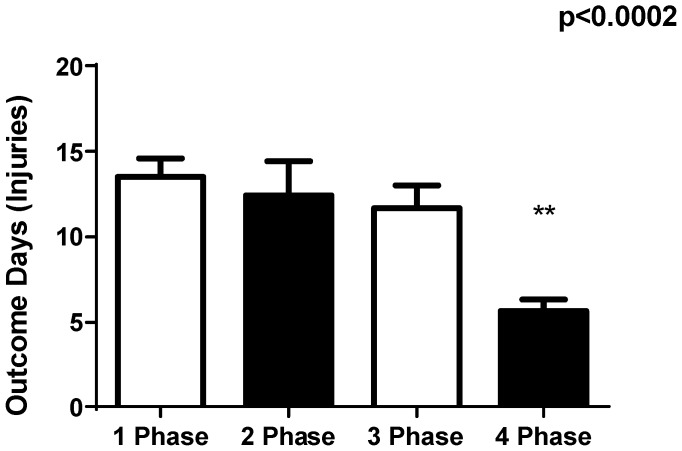
The total number of days that players were absent from training or games due to injury. ** *p* < 0.005.

Changes in physiological and functional parameters resulting from strength training are presented in Figure 1, Figure 2 and Figure 3. A significant (*p* < 0.005) change was found in BP, LPD, SP, LP, LC and SQT, Body Free Mass (kg), Body Fat (%) and Vertical Jump. 

The total number of days that players were absent from training or games due to injury decreased throughout the phases, with the injury incidence being significantly lower between phases 3 and 4 (*p* < 0.002). The greatest incidence of injuries was during the 1st and 2nd phases.

Injury days were evaluated by the Cox Regression and were based on the identification of temperature variation of at least 0.7 °C. A total of 105 measurements were diagnosed as such. Thermography was able to detect 61–69 injuries (88.40%) and to censor 44 cases: muscular (22.81%), articular (33.33%), trauma (25.00%), and tendinous (45.45%), totaling 42 events and 19 censored.

## 4. Discussion

The main findings of this study are the significant differences in injury occurrence from the beginning to the end of the year (33.3%–23.3%), and a reduction in days missed due to injury (13–6 days) across the timeline. This reduction showed that IRT mapping and ST program result in a general reduction in number of injuries and that exercise intervention programs are followed by a general reduction in these injuries [10,11,12,13]. The period of lower injury and outcome days was the fourth phase, which showed a higher index of vertical jump, squat and leg press in U-20 soccer athletes. The significance of this study is reinforced be the fact that all 26 U-20 soccer players followed the same ST program over the entire 1-year competitive period as they were trained by the same coach. Moreover, except for changes in the injury treatment program, all players were trained similarly.

These results suggest that performing ST sessions on alternate days is effective for improving muscle strength and reducing injury occurrence and incidence [11,15,20,21,22]. To the best of our knowledge, there are no studies that have investigated the impact of a one-year periodized strength training program on the occurrence and re-occurrence of injury in under 20-year-old (U-20) professional soccer players.

Strength training exercises in soccer athletes have demonstrated effectiveness in terms of increasing both concentric and eccentric strength developing higher maximal muscle torques that can respond to high-intensity strength activity and in improving initial acceleration and change-of-direction capacity in activities such as kicking and sprinting [20,21]. Athletes with higher strengths have been shown to perform better during numerous physical performance abilities and injury incidence [21,23,24]. This, therefore, highlights the potential importance that the strength has in the role of athletic development. Subsequently, athletes with high strength also provide the delay of fatigue experienced in a match, which may also contribute to a decrease in injuries and re-injuries [6,9,16]. Strength training promotes increased H^+^ (hydrogen ion) regulation and buffering capacity, and repeated sprint ability providing the enhance de physical performance [21,24]. Furthermore, our group have identified greater relative lower body strength and power as potential moderators of subsequent injury risk [17]. This reinforces the relevance of the principle of specificity in strength training for soccer prevents injuries.

In the present study, the rate of re-injury was substantially lower (15%) than other studies [4,6,9,14], with most injuries being strains and sprains. The present work credits 6% of the reported injuries to re-injuries, 46% being either strains or sprains to regions of the lower extremity. Still, one might speculate that improvements in strength and constant mapping of IRT before returning to team training and match play might reduce the risk of re-injury even more [2,4,6,14]. Re-injuries caused longer absence than non-re-injuries and required more staff attention; in this respect, the strength training was very effective in this study improving performance and assisting in injury prevention and recuperation [6,9,15].

The relation between the severity of an injury and the number of potential missed competitive matches is a marker when analyzing the impact of that injury to a club [1,2,6]. In our study, approximately 2.0 matches were missed per injury, with 68% of the injuries leading to a minimum of one missed match. Major injuries represented 59% of those reported in the present study and slight injuries represented 0.5%. The mean number of days lost per injury initially was 12, based on the incidence of injuries per 1st and 2nd phases, and decreased to six in the 4th phase, with the mean number of injuries per phase was 17 events. Player to player contact injury mechanisms including tackling, being tackled and collisions contributed for 30% of all injuries reported in the study.

It has previously been suggested that the predominance of injuries to the dominant side is due to that side being more commonly involved when tackling or being tackled [3]. The higher number of recorded strains accounted to the greater level of observed thigh injuries, as 81% of these types of injuries were muscular strains, with 67% of the thigh strains being to the posterior aspect rather than the anterior (*p* < 0.01) in the present study [2,6]. In addition, our results clearly show that the locations of many subsequent injuries are significantly biased towards the locality of the preceding injury episode.

In the current study, in IRT analyses, a total of nine athletes manifested symptoms of regional overuse symptoms. Symptomatic athletes had a mean side-to-side temperature difference of 1.4 °C (±0.58 °C). The normal temperature range of the eight non-injured athletes showed a side-to-side variation of 0.3 °C (±0.61 °C). A total of seven injured athletes reported pain, while the others were asymptomatic at that time. However, physical examination of these injured athletes revealed that this hyperthermia was associated with a low threshold for pressure pain, as previously described in the literature [15]. In this sense, our results showed that early detection and a subsequent early therapy intervention program can reduce the severity of symptoms. Furthermore, the detection of risk athletes with (RT) makes it possible to adjust their returning training program [24].

Lastly, it is worthy to mention that, despite the fact that IRT indicates the location of the injury, it cannot precisely reflect the respective etiology. Therefore, complementary exams—such as X-Ray, Computed Tomography (CT) and Magnetic Resonance Imaging (MRI)—might be required.

## 5. Conclusions

There are several implications regarding our study, as injury risk in the Brazilian U-20 soccer league is high, especially during matches. Performing ST sessions on alternate days is effective for improving muscle strength and reducing injury occurrence and incidence. Regarding this fact, preventive IRT mapping should focus on the most common diagnoses, namely, muscle/tendon injuries of the lower extremities. Initially, 27% of the players sustained multiple injuries, some up to three or more injuries and IRT and ST was efficient for reducing outcome days for injury and re-injury treatment. Our results indicate that a more intensive program of IRT care may be a sound investment for the clubs and for enhancing soccer players’ performance capacity. Coaches should aim to expose players to strength training regimens that aim to improve power and explosive qualities to best moderate injury risk.

## Figures and Tables

**Table 1:** Operational definitions used in the study.

Operational Definitions Used in the Study	
Training session	Physical activity under supervision
Match	Competitive or friendly match against another team
Injury	Player unable to participate in a match or training session
Muscular injuries	Muscle strains; muscle rupture; muscle pain
Joint injuries	Ligament sprains; overuse sprains
Trauma injuries	Collisions; contusions; concussions
Tendinous Injuries	Tendinitis; tendinosis
Injury incidence	Number of injuries per 100 h

**Table 2:** Number and location of injuries.

Local	Event	Injury (%)	Injury Incidence
thigh	16	23.19	0.16
ankle	15	21.74	0.15
knee	12	17.39	0.12
calf	9	13.04	0.09
shoulder	3	4.35	0.03
hip	6	8.69	0.06
hand	1	1.45	0.01
clavicle	3	4.35	0.03
lumbar spine	4	5.80	0.04
total	69	100	

**Table 3:** Type of injuries.

Type	Event	Injury (%)	Injury Incidence
grade 1	41	59.42	0.41
grade 2	24	34.78	0.24
grade 3	4	5.80	0.04
total	69	100	

**Table 4:** Local of injuries.

Classification	Event	Injury (%)	Injury Incidence
muscle	23	33.33	0.23
articular	23	33.33	0.23
trauma	15	21.74	0.15
tendinous	8	11.60	0.08
total	69	100	

**Table 5:** Training phases and injuries.

Phase	Event	Injury (%)	Injury Incidence
1	23	33.33	0.23
2	20	28.99	0.23
3	12	17.39	0.15
4	14	20.29	0.08
total	69	100	
